# Burns results in profound muscle protein wasting in Sprague Dawley rats that is not resolved using the lipolysis inhibitor, acipimox

**DOI:** 10.1371/journal.pone.0323640

**Published:** 2025-05-20

**Authors:** Emre Vardarli, Nisha Bhattarai, Amina El Ayadi, Anesh Prasai, Victoria G. Rontoyanni, Doaa Reda Abdelrahman, Andrew J. Murton

**Affiliations:** 1 Graduate School of Biomedical Sciences, The University of Texas Medical Branch, Galveston, Texas, United States of America; 2 Department of Surgery, The University of Texas Medical Branch, Galveston, Texas, United States of America; 3 Sealy Center on Aging, The University of Texas Medical Branch, Galveston, Texas, United States of America; 4 Shriners Children’s Texas, Galveston, Texas, United States of America; Vignan Pharmacy College, INDIA

## Abstract

**Introduction:**

Major burns results in the rapid and profound accumulation of lipid in peripheral tissues, but its impact on muscle metabolic function is unclear. Given previous reports demonstrating that lipid oversupply compromises processes instrumental in the maintenance of muscle protein balance, we hypothesize that burn-induced lipid accumulation contributes to the loss of muscle mass with thermal injury.

**Methods:**

To investigate this further, 48 male Sprague Dawley rats were randomized to undergo either a 60% total body surface area burn or sham procedure. To elucidate the impact of burn-induced lipid accumulation, animals were further subdivided to receive either acipimox (50 mg.kg^−1^ b.w.), a lipolysis inhibitor administered to deplete intramuscular lipids, or vehicle (PBS), daily for 7 days. Throughout, animals received deuterated water to permit the determination of muscle protein kinetics.

**Results:**

Compared to sham animals, burn injury resulted in a 12% loss of gastrocnemius muscle mass (P < 0.001), paralleled by a 30 and 40 increase in the fractional synthetic and breakdown rates of gastrocnemius mixed proteins (P < 0.01), respectively, culminating in a 2-fold decline in net muscle protein (P < 0.01). Contrary to expectations, burns had no impact on muscle triglyceride content, while acipimox treatment failed to protect muscle mass, impact muscle triglyceride concentrations, or muscle protein kinetics.

**Conclusions:**

In a rodent model of burns, the loss of muscle mass primarily occurs due to the acceleration of muscle proteolysis, independent of any change in muscle lipid content.

## Introduction

Major burns, encompassing greater than 20% of the body surface area, are associated with the rapid and devastating loss of skeletal muscle mass [1]. While great strides have been made in improving the survivability of major burns, persistent challenges remain in preventing muscle cachexia and subsequent frailty. Burn survivors often require protracted periods of rehabilitation to restore muscle mass and function to a level that permits a return to normal daily living. Indeed, longitudinal studies have demonstrated that individuals following major burn trauma experience an ~ 30% decline in lean mass that is still evident three years post injury [2]. Thus, it is critical to understand the underlying mechanisms driving muscle catabolism caused by burns and develop efficacious treatment interventions.

A potential contributor to the loss of muscle mass with major burns is the rapid ectopic deposition of lipid that occurs, a consequence of persistent catecholamine-driven adipose tissue lipolysis [3] and the diminished oxidation of lipid species [4]. These diametrically opposed events results in the diversion of lipid towards cellular storage and are thought to underpin the reports of increased intramuscular lipids seen in both burn patients [5] and rodent models of burns [6]. We contend that the oversupply of lipid to muscle is detrimental to the tissue’s overall metabolic function and contributes to the pronounced muscle catabolism seen with burns. In support, we have previously observed that the ability of human muscle to effectively utilize amino acids to synthesize contractile proteins is diminished with increasing adiposity [7]. Moreover, artificially increasing lipid availability via the intravenous infusion of a lipid emulsion, approximately doubling plasma non-esterified fatty acid concentrations, results in the rapid blunting of muscle protein anabolism in response to hyperaminoacidemia [8], a process essential in the maintenance of muscle mass and quality (see [9]). Viewed in conjunction with evidence that increased lipid availability can induce myotube atrophy and upregulate mediators of muscle proteolysis [10], it stands to reason that burn-induced ectopic lipid accumulation could compromise the ability to maintain or restore muscle mass.

In the current study, we investigated the influence of burn-induced lipid oversupply on muscle protein kinetics and catabolism. We employ acipimox, a niacin derivative known for its strong anti-lipolytic activity, to elucidate the effects of lipid accumulation on the turnover rates of muscle protein in a rodent model with major burns. We hypothesize that acipimox will prevent burn-induced lipid accumulation in muscle, blunting the net loss of muscle protein, and culminating in the protection of muscle mass to thermal injury.

## Materials and methods

### Animals

Male Sprague Dawley rats (n = 48), approximately 4 months of age and with a body weight no greater than 50 g different at arrival, were purchased from Charles River, USA, and housed in the animal facilities of the University of Texas Medical Branch. The below animal protocol was approved in advance by the Institution’s Animal Care and Use Committee (REF: 1812094) and all animals were inspected on arrival and found to be in good health. Following arrival, animals were acclimatized to their new environment for a minimum of one week before being randomly assigned to receive either an ~ 60% total body-surface area (TBSA) scald burn or sham procedure (n = 24/group). The thermal injury was performed using an approach [11] that is known to induce a full-thickness burn without injuring the underlying tissues, and elicit a robust pro-inflammatory and hypermetabolic response [12,13], mimicking the response seen to major burns in patients. In short, animals receiving a burn injury were provided with pre-emptive analgesia (buprenorphine 0.05 mg.kg^−1^ b.w. s.c.), and anesthetized with isoflurane inhalation anesthetic, with the dorsum and abdomen of the animal shaved with electrical clippers. Afterwards, the dorsum of the animal was immersed in 96–98 °C water for 10 seconds, and the abdomen for 2 seconds, with timing confirmed via the use of a digital stopwatch and a trained observer. A large volume water bath was used to ensure the maintenance of the water temperature throughout the procedure, and the temperature confirmed via use of an inbuilt digital thermometer. A custom-made plastic mold containing an open window in which animals were secured during the procedure, controlled the surface area of the skin in direct contact with the scalding water. The adequacy of anesthesia was confirmed prior to the burn procedure by testing for the absence of the pedal withdrawal reflex and the lack of eye blink reflex. While anesthetized, the respiratory rate and depth was monitored visually, along with adequate oxygenation via examination of the color of the ears, tail, and gums, with anesthesia depth adjusted accordingly.

Lactated Ringer’s solution was provided via i.p. injection (30 ml.kg^−1^ b.w.) to fluid resuscitate the animals. The need for fluid resuscitation is two-fold: firstly, to prevent direct thermal damage to the spinal column from the burn procedure and secondly to replace the loss of intravascular volume that occurs with major burns, thereby maintaining tissue perfusion and preventing end-organ hypoperfusion and ischemia. Sham animals were treated in the same manner but were submerged in lukewarm water and did not receive the resuscitation fluid. To measure chronic muscle protein synthesis rates, we employed an established protocol using heavy water as a metabolic label, as we’ve described previously [14]. To achieve this, all animals received a 10 ml.kg^−1^ b.w. i.p. bolus of heavy water (70% atom percent excess), administered immediately post-burn/sham procedure. Afterwards, the drinking water was supplemented with 2% v/v of the same heavy water with animals singly housed in wire-bottom cages in an environmental temperature of 22–24 °C and under a 12-hour light-dark cycle. All animals were provided with unrestricted access to standard chow (18% protein; Global 2018, Harlan Teklad, USA) and labelled water. Analgesics (buprenorphine as above, administered up to every 8 hours) and topical antibiotics (neomycin, polymyxin B and bacitracin) were administered in response to the presence of either pain or loss of skin barrier integrity, respectively. Throughout the acclimatization period and post-procedure, animals were checked daily by both veterinary staff and the Investigators to ensure the health and welfare of the animals. The adequacy of analgesia post-burn procedure was determined twice daily via the combined use of the Rat Grimace Scale, an assessment of the animal’s behavior, and inspection of the injury. Animals showing signs of pain or distress were administered buprenorphine for analgesia (0.05 mg.kg^−1^ b.w. s.c.), repeated up to every 8 hours until resolution of pain symptoms. One animal with a burn injury treated with acipimox was euthanized prior to day 7 due to the presence of pain that could not be resolved with buprenorphine treatment.

Following completion of the burn or sham procedure, animals were randomized to receive either acipimox (50 mg.kg b.w.^−1^.day^−1^) or vehicle (phosphate buffered saline) administered daily for 7 days by oral gavage (n = 12/group). The dose and route of administration of acipimox were selected based on previous reports demonstrating that 50 mg.kg^−1^ per day for 7 days was effective at reducing burn-induced hepatic lipid accumulation in mice [15]. On the seventh day, animals were further subdivided into two groups in an attempt to examine the acute metabolic response to key nutritional cues, as we have described previously [14]. Crucially, 7 days post-burn coincides with a time when ectopic lipid accumulation [15] and muscle protein catabolism [16] have previously been reported. Following a 6 hour fast, animals received either a combination of leucine (1.35 g.kg b.w.^−1^ by oral gavage in PBS) and insulin (100 mU Actrarapid by i.p.), or vehicle (PBS). All animals received a glucose analogue (2-deoxyglucose, 0.5 g.kg b.w.^−1^ in PBS by i.p.) and ^2^H_5_-phenylalalnine (50 mg.kg b.w.^−1^ in PBS by tail vein injection), to permit the determination of muscle protein synthesis and glucose uptake kinetics. Leucine, insulin, 2-deoxyglucose and ^2^H_5_-phenylalanine were all administered within 60 minutes of planned euthanasia. At the allotted time, animals were decapitated with blood collected into EDTA containing vacutainers, centrifuged, and the resultant plasma stored at -80 °C prior to analysis. The gastrocnemius, tibialis anterior (TA), extensor digitorum longus (EDL), and soleus hindlimb muscles were rapidly excised, weighed, and snap frozen in liquid nitrogen for subsequent analysis.

On subsequent analysis, it was determined that the leucine oral gavage inconsistently increased plasma leucine concentrations, a likely consequence of the poor solubility of leucine and the limited volume of liquid that can be administered via the oral gavage route. Given the solubility of leucine in water (22.4 g/l at 20 °C), fully dissolving the allotted leucine (1.35 g.kg bw^−1^) would have required an oral gavage volume of 60 ml.kg b.w.^−1^, beyond the volume permitted by most Institutional Animal Care and Use Committees. Given that consistent hyperleucinemia was not observed with exogenous leucine treatment, it was decided that the data obtained from the acute metabolic assessment, performed in the final hour of the study, would not be considered further. The impact on the chronic assessment of muscle protein turnover, performed over a 7-day (168 hour) window, would be expected to be negligible.

### Quantification of gastrocnemius triglyceride concentrations

Gastrocnemius triglyceride (TAG) concentrations were quantified using a commercially available kit (Catalog # MAK266, Millipore-Sigma, USA), according to the manufacturer’s instructions. In short, ~ 20 mg of muscle was homogenized in 5% triton buffer, initially with aid of a bead beater (15 sec x 8 cycles) before being further homogenization with a motorized pestle grinder. Afterwards, samples were heated for 5 minutes at 90 °C before being centrifuged at 18,000 g for 2 minutes, with the resultant supernatant diluted 5-fold in the proprietary assay buffer. Fifty µl of the resultant solution was added to a 96-well plate in duplicate, incubated for 60 min at room temperature, and the absorbance of the color product read at 570 nm with aid of a microplate spectrophotometer. Included standards were used to quantify muscle TAG concentrations.

### Determination of chronic muscle protein turnover rates

Approximately 30 mg of gastrocnemius muscle was processed for the determination of deuterated-alanine enrichment of mixed muscle proteins as we’ve described previously [14,17,18]. In short, tissue was homogenized in 10% perchloric acid, centrifuged, and the pelleted proteins serially washed in 2% perchloric acid, absolute ethanol, and ethyl ether. After further centrifugation, the pelleted protein was hydrolyzed overnight at 100 °C in 6N HCl, before the hydrolyzed proteins were purified by cation chromatography, with amino acids eluted through the addition of 2N ammonium hydroxide. Eluted samples were dried overnight by vacuum centrifugation before being derivatized via the addition of 80 µl of acetonitrile:N-tert-butyldimethylsilyl-N-methyltrifluoroacetamide (1:1) and heating at 95 °C for 40 minutes to produce tert-butyldimethylsilyl derivatives. Samples were analyzed on a gas chromatography-pyrolysis-isotope ratio mass spectrometer (Trace 1310 GC coupled to a Delta V IRMS via a high-temperature thermal conversion oven; ThermoFisher Scientific), as described previously [17,18].

Body water deuterium enrichment was measured in plasma collected at euthanasia. To accomplish this, plasma was diluted 10-fold in water and 200 μl transferred to an exetainer vial containing a platinum catalyst, and flushed with 2% helium at RT by using a Gasbench II (Thermo Scientific) online gas preparation system. After the sample was given time to equilibrate, the sample was analyzed by IRMS (Delta V, ThermoFisher) to determine deuterium enrichment of body water. Intracellular free alanine enrichment was estimated by multiplying plasma deuterium enrichment by 3.7, to account for the known affinity of deuterium for hydrogen via substitution in the alanine molecule [19].

The gain of muscle protein content occurs when the rate at which muscle proteins are synthesized persistently exceeds the rate at which they are degraded. This is determined experimentally by establishing the fractional synthetic and breakdown rates of muscle proteins, with the difference between the two rates used to establish the change in net muscle protein balance over the labeling period. Muscle protein fractional synthetic rate (FSR; equation 1), fractional breakdown rate (FBR; equation 2), and net balance (NB; equation 3) were determined over the 7-day period of heavy water labelling using equations proposed by Miller and colleagues [20], where P0 and P(t) represent the protein mass of the tissue at time zero and at euthanasia, respectively. For the former, this was taken as the protein mass seen in the sham/vehicle treated animals, with protein concentrations determined using 10 mg of tissue homogenized in ice-cold PBS and analyzed using a commercial bicinchoninic acid assay kit (Pierce, USA). In the below, E(t) and E* represent alanine deuterium enrichment in the bound and free pools, respectively, while t represented the length of heavy water labelling, in hours.


$$\mathrm{\backslash [}FSR=\;\frac{100}{t}\frac{1}{P_{0}}[P_{0}-P(t)\left(1-\;\frac{E(t)}{E^{*}}\right)]\mathrm{\backslash ]}$$ (1)

$$\mathrm{\backslash [}FBR=\;\left(\frac{-1}{t}\right)\ln\left[\left(1-\frac{E(t)}{E^{*}}\right)\frac{P(t)}{P_{0}}\right]x\;100\mathrm{\backslash ]}$$ (2)

NB=FSR−FBR (3)

### Statistics

A three-way ANOVA was performed to determine differences in body weight (injury x acipimox treatment x time). When a significant interaction was observed, a series of two-way-ANOVAs were performed to explore the differences between factors (injury x acipimox treatment; injury x time; acipimox treatment x time). Insulin and leucine administration had no impact on any of the outcome measures examined and therefore was not considered as a factor in the analysis. Two-way-ANOVAs (injury x acipimox treatment) were performed to examine differences between factors in outcome measures, with Tukey’s post-hoc analysis used to locate differences amongst groups when a significant interaction between terms was observed. Statistical analyses were performed using GraphPad Prism version 6 (GraphPad Software, La Jolla, CA). Outliers were identified via the Grubbs’ test and removed from further consideration (P < 0.05). Statistical significance was accepted when P < 0.05, with data reported as mean ± SEM.

## Results

**Fig 1:**
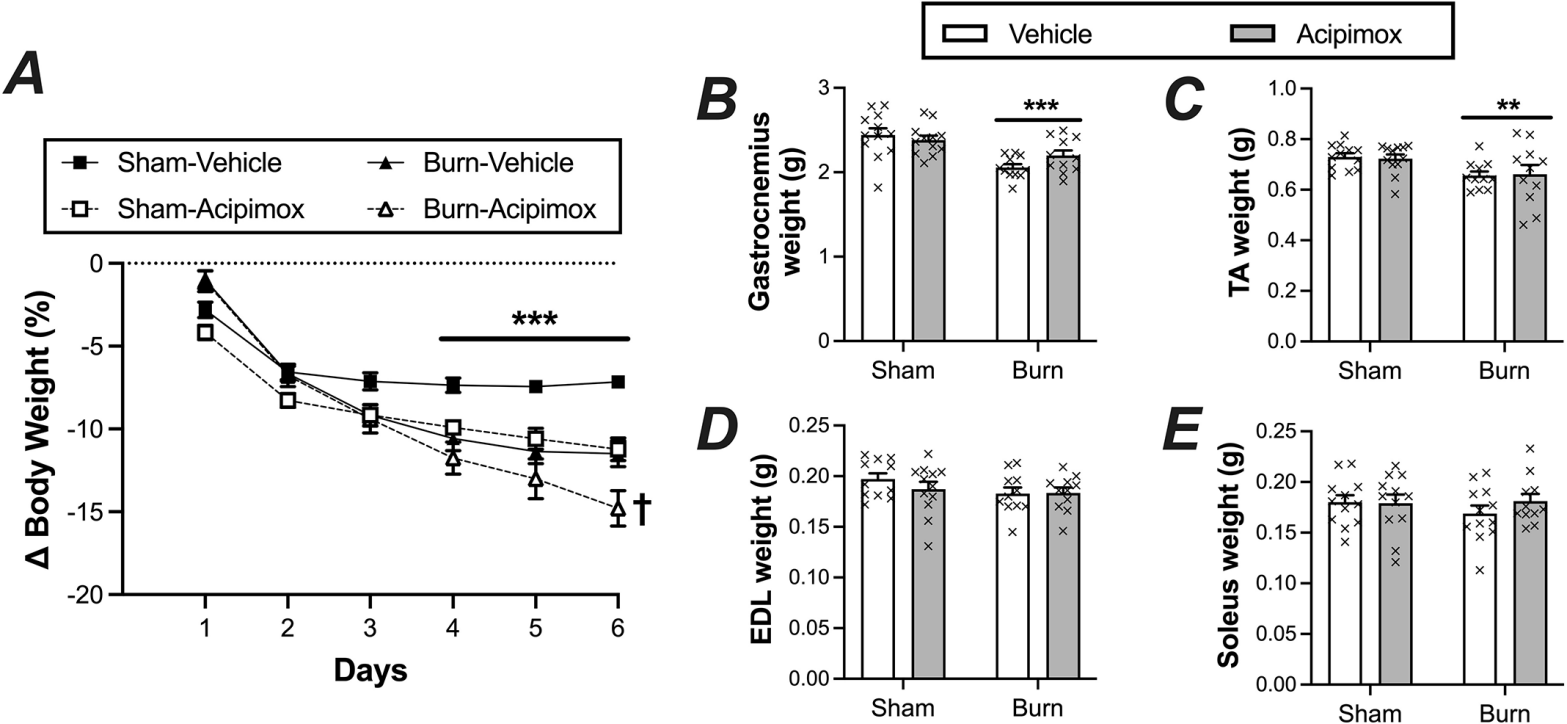
Impact of a ~60% TBSA burn and acipimox treatment on body and muscle weight. (A) Daily change in body weight; *** P < 0.001: sham-acipimox, burn-vehicle and burn acipimox significantly different than sham-vehicle. † P < 0.05: burn-acipimox significantly lower vs. other groups at day 6. Wet weight of gastrocnemius (B), tibialis anterior (C), extensor digitorum longus (D), and soleus (E) following 7 days post burn and/or acipimox treatment. Data represents means ± SEM. ** P < 0.01 and *** P < 0.001: significant main effect of burns. No significant main effect of acipimox or an interaction between main effects was observed.

Baseline body weight was not significantly different between groups (520 ± 3 g) and all animals lost ~ 7% body weight over the first 2 days following the burn or sham procedure (Fig 1A, main effect of time, P < 0.0001). Body weight in the sham-vehicle group stabilized thereafter, but a continual decline in body weight was observed in animals receiving acipimox and/or subjected to burn injury which persisted for the entirety of the study (main effects of burn and acipimox both P < 0.001). Notably, weight loss was more pronounced in the burn-acipimox group than animals receiving acipimox treatment or subjected to burn injury alone (burn x acipimox interaction; P = 0.008), with a significant difference seen between groups at day 6 (P < 0.02).

**Fig 2:**
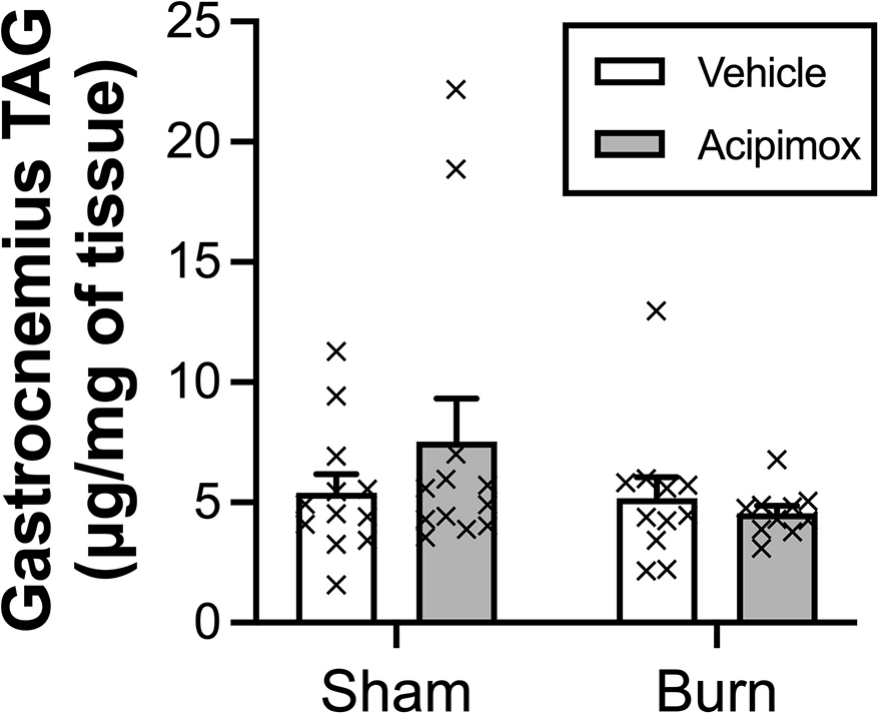
Gastrocnemius TAG concentration in response to a 60% TBSA burn and/or acipimox treatment. No significant impact of burn injury or acipimox treatment on gastrocnemius TAG concentration was observed. Values represent mean ± SEM.

Mirroring changes in body weight, the wet weight of the gastrocnemius and TA muscles were approximately 12% and 7%, lower in burn versus sham animals, respectively (Fig 1B and 1C; main effect of burn injury P < 0.001 and P < 0.01, respectively). In contrast, burn injury did not affect either EDL or soleus muscle weight (Fig 1D and 1E), while acipimox treatment did not affect the wet weight of any of the tissues examined. Given that the greatest loss of muscle weight was observed in the gastrocnemius, subsequent determination of protein kinetics and triglyceride concentrations were performed in the tissue. However, contrary to expectations, neither burn injury nor acipimox treatment had any impact on gastrocnemius triglyceride (TAG) concentrations (Fig 2), with equivalent concentrations seen irrespective of group assignment.

**Fig 3:**
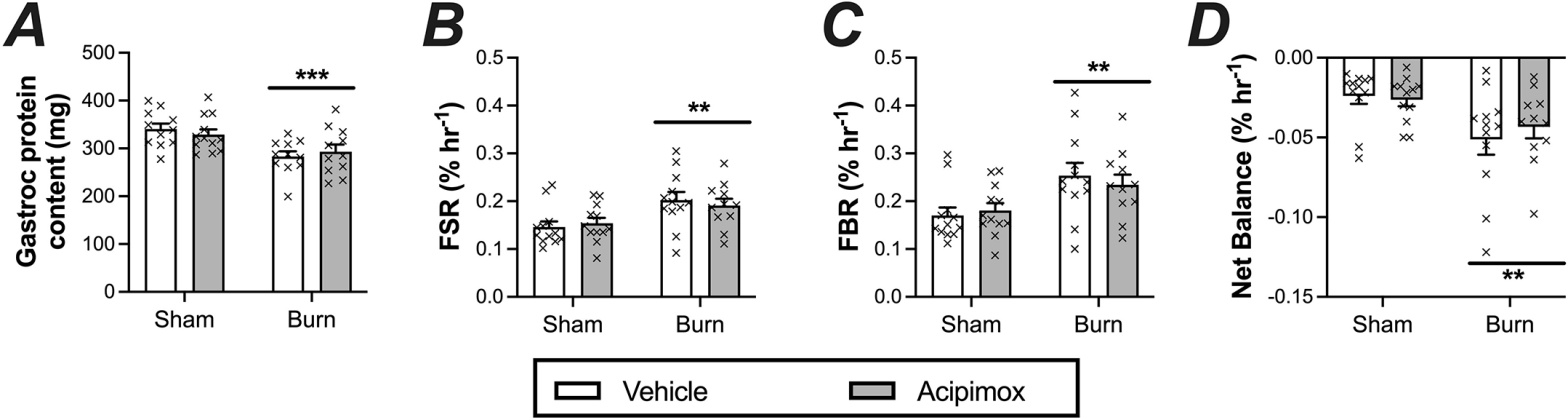
Effects of major burns and acipimox treatment on muscle protein kinetics. (A) Gastrocnemius protein mass, (B) mixed muscle protein fractional synthetic rate, (C) fractional breakdown rate, and (D) net protein balance in response to burn injury and daily acipimox treatment. Protein kinetics assessed over a 7-day window following burn injury using heavy water labelling. Data represents means ± SEM. ** P < 0.01; *** P < 0.001: significant main effect of burns. No significant main effect of acipimox or an interaction between main effects was observed.

Total gastrocnemius protein content, paralleling the observed decline in muscle mass, was significantly lower in burn versus sham injured animals (Fig 3A; main effect: P < 0.001), and was unaffected by acipimox treatment. Consequently, habitual muscle protein turnover rates, as determined by examining deuterated alanine incorporation into bound protein, was calculated using non-steady state equations accounting for the loss of muscle protein content [20]. Utilizing this approach and mimicking observations reported in patients with major burns [21], both the fractional synthetic and breakdown rates of mixed muscle proteins was significantly higher in burn compared to sham animals, increasing by 30 and 40%, respectively (Fig 3B and 3C, main effect of burn injury, P < 0.002). The discordance between the rate at which muscle proteins are synthesized and degraded resulted in a in 2-fold greater negative net protein balance in burn versus sham animals (Fig 3D; main effect of burns: P = 0.002). Acipimox treatment had no impact on any aspect of gastrocnemius protein kinetics examined.

## Discussion

Major burns results in the profound and rapid loss of muscle mass, impacting an individual’s quality of life and culminating in protracted recoveries [2]. Despite this, effective treatment solutions that can blunt the loss of skeletal muscle mass following thermal injury remain elusive. We postulated that the induction of ectopic lipid deposition post-injury acts as a major contributor to the loss of muscle protein content, suppressing the synthesis of muscle proteins, while accelerating muscle proteolysis. We demonstrate for the first time that rat muscle experiences the same protein metabolic response to burn injury as humans but fails to display evidence of muscle lipid accumulation or benefit from the provision of acipimox, a potent inhibitor of lipolysis. Given the marked induction of muscle catabolism in the absence of muscle lipid accumulation, our results suggest that alternative mechanisms are responsible for the bulk of the atrophy observed in response to major burns.

Notably, major burn injury led to a significant and progressive decrease in body weight, amounting to approximately 13% over a one-week period. Furthermore, the trauma resulted in substantial skeletal muscle wasting of predominantly fast-twitch muscles (gastrocnemius and tibialis anterior, but notably not the EDL), while slow-twitch muscles (soleus) appeared unaffected, mirroring the fiber-type specific loss of muscle mass seen in many other diseases characterized by cachexia. Glucocorticoids, which significantly increase following burns, have been implicated in burn-induced muscle wasting [22,23] and induce muscle wasting primarily in fast-twitch muscles as opposed to slow-twitch muscles [22,24,25], providing one possible explanation for the fiber-type dependent atrophy observed in the current study. Moreover, given the animals in our study remained mobile throughout, in stark contrast to burn patients who are typically bed-ridden following the acute injury, it is plausible that this relatively limited mobility staved off catabolism of the slow-twitch muscles given their sensitivity to loading [26].

While acipimox treatment has been previously shown to attenuate weight loss in mice subjected to major burns [15], we observed an acceleration in the decline of body mass with administration of the niacin derivative, irrespective of whether animals had received a thermal injury. Notably, while this discrepancy between trials was not due to a difference in dosing strategy, with both employing the same dose, route, and length of administration, it is permissible that the use of mice as a model, or smaller burn size (30% TBSA), could underpin the differences between the study of Barayan and colleagues, and that of the data reported here. Their observation of an almost doubling of food intake in burned mice with acipimox treatment compared to control animals, compared to our observations of weight loss with acipimox treatment irrespective of injury status, suggests that a difference between the two animal models underpins the divergent results observed between studies. However, despite our observations of weight loss with acipimox administration, the drug appeared to have no detrimental impact on muscle mass. Given that acipimox has been suggested as a potential treatment strategy to alleviate the hypermetabolic response to burns [15,27], our findings nonetheless suggest that the impact of the drug on body weight warrants further investigation.

Despite validating the catabolic nature of the rat burn model, it appears apparent that the current model does not display the same excessive accumulation of muscle lipids as has been seen in patients and rodents following major burns [6,15]. Contrary to expectations, in the current study, major burns had no impact on muscle TAG concentration. We postulate that methodological differences between studies could be responsible for the failure to observe ectopic lipid deposition in the current trial. In particular, the proximity of the burn to the examined tissue could be critical. The tissues immediately beneath a burn are affected to a greater extent than tissues located distally [28–30]. Minor and localized burns (3–5% TBSA), located on a hindlimb of rodents have been shown to promote a substantial 9-fold increase in intracellular lipid content in the muscles immediately under the burn site [6]. The mechanisms responsible for the intensified effects of local burns have yet to be thoroughly explored. However, it has been argued that localized inflammation and lipid peroxidation are considerably greater in tissues underlying the burn, culminating in the increased breakdown and release of membrane lipids, contributing to intracellular lipid accumulation independent of adipose tissue lipolysis [31,32]. In support of this argument, Kurdle and colleagues demonstrated via magnetic resonance imaging that there is a tendency for lipids to preferentially accumulate in areas close to the burn site [33]. Collectively, these observations suggest that the tissue location with respect to the burn site plays a role in determining the extent of lipid accumulation in burns and could explain differences between studies. Consequently, further research is warranted to elucidate how burns differentially alter lipid metabolism and its deposition in tissues located near and distal to the burn site.

Alternative explanations for the failure to observe ectopic lipid accumulation in the current trial could be due to the timing of when tissue was collected or the decision to focus on muscle triglyceride content. Given that lipids are primarily stored in muscle as triglycerides [34], their quantification was prioritized, but nevertheless it remains plausible that other potent lipid species such as diacylglycerides were increased in muscle with burn injury. Conversely, in Sprague-Dawley rats, contrasting profiles of tissue lipid accumulation have been observed during the recovery period. This suggests that a temporal pattern of lipid accumulation could exist in the initial days following the trauma versus later stages. In support, Jayaraman et al. examined the temporal changes in the expression of metabolic and inflammatory genes of the liver at four different time points (1, 2, 4, and 7 days) following a 20% TBSA burn in Sprague-Dawley rats [35]. They observed that liver lipid concentrations increased during the first 24 hours, but progressively decreased thereafter. While temporal changes in muscle lipid accumulation post burns has not been studied in detail, it is possible that changes in muscle TAG concentrations had normalized by 7 days post-injury. Regardless, the failure to observe a demonstrable benefit of acipimox treatment on chronic muscle protein metabolism over the 7-day period examined suggests that burn-induced muscle lipid accumulation had a negligible effect on protein metabolism, even if an early transient increase in tissue lipid content was missed.

Habitual muscle protein kinetics, capturing the cumulative changes in both protein synthesis and breakdown rates over a seven-day window was employed to determine the burn-induced alterations in muscle protein metabolism that underpins the muscle wasting observed. Our findings revealed that major burns in rats led to a state of hyper-protein metabolism, closely resembling changes observed in burn patients [21,36]. In particular, we observed the signature elevation of both muscle protein FSR and FBR, a characteristic of burns, but with the protein FBR far exceeded the rise in protein FSR, culminating in a reduction of net protein balance and the loss of muscle mass. These findings reinforce proteolysis as the principal driver of muscle wasting in both burn patients and rodent models and reinforces the utility of the animal model for research focused on devising therapeutic strategies to overcome burn-induce muscle catabolism. In addition, considering that muscle lipid concentrations were not increased following burn injury, our findings indicate that major burns impair muscle protein metabolism and result in muscle wasting independent of any purported accumulation of lipids. Moreover, we demonstrate that acipimox treatment has no beneficial impact on muscle protein metabolism, despite its purported benefits at reducing burn-induced hypermetabolism [15].

Limitations of the current trial were the examination of a single time point and the inability to record daily food intake. The latter was hindered by caging apparatus that precluded the accurate determination of food intake, a parameter that could have been affected by either the burn injury or acipimox treatment. In the absence of data on the impact of injury and acipimox on food consumption, it remains permissible that decreased food intake explains a component of the loss of body weight and muscle mass that is observed post-burns. An additional limitation was our focus on a single muscle (the gastrocnemius) for detailed metabolic analysis. The gastrocnemius was selected based on the presence of burn-induced atrophy and its large mass permitting the assessment of protein turnover and lipid abundance in the same muscle. However, it’s feasible that the impact of burns and acipimox treatment on muscle protein turnover and lipid deposition differ between muscles of different fiber type compositions. Irrespective of these limitations, it can be concluded that the loss of muscle mass with major burns occurs primarily due to the acceleration of muscle proteolysis, independent of any change in muscle lipid content. Furthermore, we demonstrate that the use of acipimox, a potent lipolysis inhibitor fails to improve muscle protein balance and in contrary, exacerbates burn-induced weight loss, a concern in any future therapeutic use of the drug in burn patients.
